# ATG8 Is Essential Specifically for an Autophagy-Independent Function in Apicoplast Biogenesis in Blood-Stage Malaria Parasites

**DOI:** 10.1128/mBio.02021-17

**Published:** 2018-01-02

**Authors:** Marta Walczak, Suresh M. Ganesan, Jacquin C. Niles, Ellen Yeh

**Affiliations:** a Department of Biochemistry, Stanford Medical School, San Francisco, California, USA; b Department of Pathology, Stanford Medical School, San Francisco, California, USA; c Department of Microbiology and Immunology, Stanford Medical School, San Francisco, California, USA; d Department of Biological Engineering, Massachusetts Institute of Technology, Cambridge, Massachusetts, USA; e Chan Zuckerberg Biohub, San Francisco, California, USA; University of Pittsburgh

**Keywords:** ATG8, *Plasmodium*, apicoplast, apicoplast biogenesis, autophagy, malaria

## Abstract

*Plasmodium* parasites and related pathogens contain an essential nonphotosynthetic plastid organelle, the apicoplast, derived from secondary endosymbiosis. Intriguingly, a highly conserved eukaryotic protein, autophagy-related protein 8 (ATG8), has an autophagy-independent function in the apicoplast. Little is known about the novel apicoplast function of ATG8 and its importance in blood-stage *Plasmodium falciparum*. Using a *P. falciparum* strain in which ATG8 expression was conditionally regulated, we showed that *P. falciparum* ATG8 (*Pf*ATG8) is essential for parasite replication. Significantly, growth inhibition caused by the loss of *Pf*ATG8 was reversed by addition of isopentenyl pyrophosphate (IPP), which was previously shown to rescue apicoplast defects in *P. falciparum*. Parasites deficient in *Pf*ATG8, but whose growth was rescued by IPP, had lost their apicoplast. We designed a suite of functional assays, including a new fluorescence *in situ* hybridization (FISH) method for detection of the low-copy-number apicoplast genome, to interrogate specific steps in apicoplast biogenesis and detect apicoplast defects which preceded the block in parasite replication. Though protein import and membrane expansion of the apicoplast were unaffected, the apicoplast was not inherited by daughter parasites. Our findings demonstrate that, though multiple autophagy-dependent and independent functions have been proposed for *Pf*ATG8, only its role in apicoplast biogenesis is essential in blood-stage parasites. We propose that *Pf*ATG8 is required for fission or segregation of the apicoplast during parasite replication.

## IMPORTANCE

*Plasmodium* parasites, which cause malaria, and related apicomplexan parasites are important human and veterinary pathogens. They are evolutionarily distant from traditional model organisms and possess a unique plastid organelle, the apicoplast, acquired by an unusual eukaryote-eukaryote endosymbiosis which established novel protein/lipid import and organelle inheritance pathways in the parasite cell. Though the apicoplast is essential for parasite survival in all stages of its life cycle, little is known about these novel biogenesis pathways. We show that malaria parasites have adapted a highly conserved protein required for macroautophagy in yeast and mammals to function specifically in apicoplast inheritance. Our finding elucidates a novel mechanism of organelle biogenesis, essential for pathogenesis, in this divergent branch of pathogenic eukaryotes.

## INTRODUCTION

*Plasmodium* (the causative agent of malaria) and other apicomplexan parasites are important human and veterinary pathogens. In addition to their biomedical significance, these protozoa represent a branch of the eukaryotic tree distinct from the well-studied model organisms that are the textbook examples of eukaryotic biology. As such, parasite biology often reveals startling differences that both highlight the diversity of eukaryotic cell biology and can potentially be leveraged for therapeutic development. A prime example of this unique biology is the nonphotosynthetic plastid organelle the apicoplast. It was acquired by an unusual secondary eukaryote-eukaryote endosymbiosis, in which an alga was engulfed by another eukaryote, forming a new secondary plastid in the host (1). Although the apicoplast has lost the photosynthetic function, it contains several metabolic pathways and is essential for parasite survival during human infection (2, 3). Despite its importance to pathogenesis, little is known about how the apicoplast coordinates its biogenesis with parasite replication.

*A priori* this unique apicomplexan organelle should have little to do with a highly conserved eukaryotic protein, autophagy-related protein 8 (ATG8). In model organisms, ATG8 plays a central role in autophagy, a conserved eukaryotic pathway for the degradation of cytoplasmic components. During autophagy, cytoplasmic cargo is sequestered in a double-membrane autophagosome which fuses with the lysosome. The ubiquitin-like ATG8 is covalently attached to phosphatidylethanolamine (PE) on the inner and outer membranes of the autophagosome (4). On the autophagosome membrane, it is required for cargo selection, *de novo* formation of the autophagosome, and lysosomal fusion and is the key marker used to identify autophagosomes (5). In fact, blood-stage *Plasmodium* parasites have been reported to accumulate ATG8^+^ vesicles that may represent autophagosomes upon amino acid starvation (6, 7), while ATG8^+^ autophagosome-like structures in liver-stage parasites are required for the turnover of invasion organelles (8).

Yet *Plasmodium* ATG8 clearly has a novel function in the apicoplast, distinct from its role in autophagy. Numerous groups independently showed that ATG8 localizes to the apicoplast in blood- and liver-stage *Plasmodium* as well as the related parasite *Toxoplasma gondii* (6, 7, 9–12). Apicoplast localization occurs throughout the parasite replication cycle and is independent of autophagy inducers and inhibitors (6, 9, 13). This function is likely important since the apicoplast is essential for parasite replication during host infection. Indeed, while yeast and mammalian ATG8 homologs are nonessential under nutrient-replete conditions (14, 15), knockdown of ATG8 in *T. gondii* leads to a block in parasite replication with defects in apicoplast biogenesis (16). Consistent with an essential function in *Plasmodium*, ATG7, a component of the ATG8 conjugation system, is essential in blood-stage *Plasmodium falciparum* (17), while ATG8 overexpression in liver-stage *Plasmodium berghei* results in nonviable parasites with apicoplast defects (8). Curiously, a pool of cytoplasmic yeast and mammalian ATG8 must be cleaved to expose the glycine residue, which is then conjugated to the autophagosome membrane (18). In contrast, the last residue of all apicomplexan ATG8 homologs is glycine and no pool of soluble *P. falciparum* ATG8 (*Pf*ATG8) is detectable, indicating that its membrane conjugation is regulated differently (6, 9).

Key questions remain. Is ATG8 required for apicoplast biogenesis in the symptomatic blood stage of *Plasmodium falciparum*? It seems likely given the essentiality of *Pf*ATG7 and the phenotypes observed in liver-stage *P. berghei* and *T. gondii* but has not been demonstrated. What is ATG8’s function in apicoplast biogenesis? The abnormal proliferation of apicoplast membranes observed in liver-stage *P. berghei* overexpressing ATG8 was attributed to its role in membrane expansion (8). Meanwhile, the association of ATG8 with vesicles containing apicoplast proteins in blood-stage *P. falciparum* suggested a role in vesicle-mediated protein import into the apicoplast (6, 7). Alternatively, *T. gondii* ATG8 (*Tg*ATG8) was proposed to mediate the interaction of the apicoplast with the centrosome (16). Since multiple autophagy-dependent and independent ATG8 functions have been proposed, does *Pf*ATG8 have other functions in the blood stage essential for parasite replication? For example, ATG8 may have a role in vesicle trafficking to the food vacuole, the lysosomal compartment for host hemoglobin digestion, which is essential for growth in red blood cells (RBCs) (7, 19–21). ATG8’s apicoplast function may be particularly challenging to unravel if other ATG8 functions are also essential.

To answer these questions, we generated a *P. falciparum* strain in which ATG8 expression was conditionally regulated. We assessed parasite replication and apicoplast defects upon ATG8 knockdown, taking advantage of a novel apicoplast chemical rescue available only in blood-stage *P. falciparum*. Not only is *Pf*ATG8 essential for blood-stage *Plasmodium* replication, but its only essential function is in apicoplast biogenesis, where it is required for apicoplast inheritance.

## RESULTS

### ATG8 is essential for blood-stage *Plasmodium* replication and apicoplast function.

**FIG 1:**
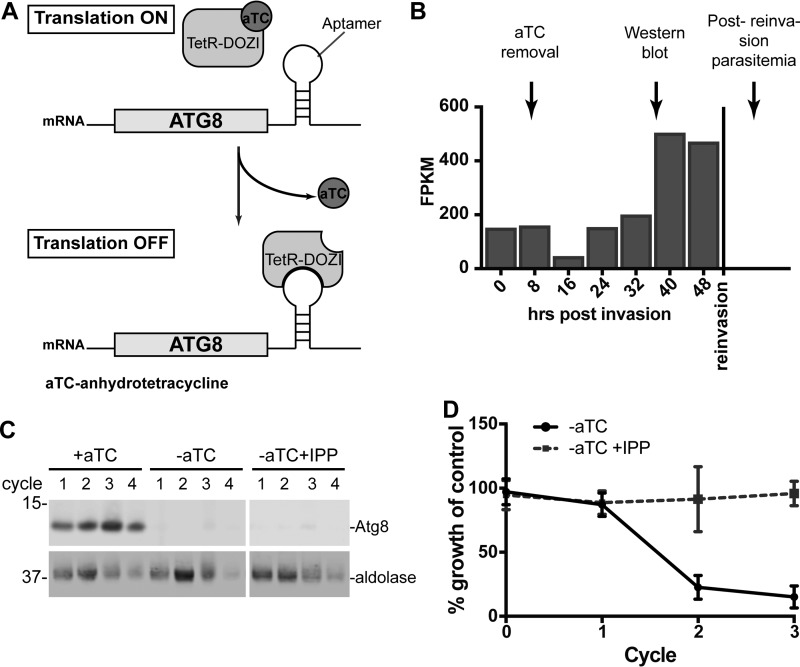
ATG8 is essential for parasite replication and apicoplast function. (A) Regulation of ATG8 expression by anhydrotetracycline (aTC)-dependent binding of TetR-DOZI repressor. (B) Timing of aTC removal and sample collection during a single replication cycle overlaid with ATG8 expression profile (75). FPKM, fragments per kilobase per million. (C) Western blot showing ATG8 knockdown in the presence or absence of IPP. Equal parasite numbers were loaded per lane. Numbers on the left are molecular masses in kilodaltons. (D) Parasitemia of ACP_L_-GFP-expressing cultures grown for 4 cycles under the indicated conditions, normalized to culture grown in the presence of aTC, i.e., expressing ATG8. Mean ± standard deviation for 3 biological replicates is shown.

To determine whether *Pf*ATG8 is essential, we generated a conditional expression strain in which the endogenous ATG8 locus was modified with a C-terminal myc tag and 3′ untranslated region (UTR) TetR-DOZI-binding aptamer sequences for regulated expression (see Fig. S1 in the supplemental material). As expected, ATG8 expression was induced in the presence of anhydrotetracycline (aTC), which disrupts the TetR-DOZI repressor-aptamer interaction (+aTC condition [Fig. 1A and C]) (22, 23). Though ATG8 was detectable by antibodies against full-length protein, it was not detectable by myc antibodies (Fig. S1), suggesting that the C terminus of ATG8 was cleaved. This cleavage may be mediated by the putative *Plasmodium* homolog of the ATG4 protease which removes C-terminal extensions on ATG8, either naturally present (in most model eukaryotes) or artificially added (11, 18, 24). Removal of aTC at the beginning of the parasite replication cycle resulted in efficient knockdown with no detectable ATG8 protein within the same cycle (Fig. 1B and C). We monitored the growth of ATG8-deficient parasites and observed a dramatic decrease in parasitemia over 2 or more replication cycles compared to control ATG8^+^ cultures (Fig. 1D). These results show that *Pf*ATG8 is essential for parasite replication in blood-stage *P. falciparum*.

10.1128/mBio.02021-17.1 FIG S1  (A) Conditional ATG8 knockdown line (ATG8-TetR) generated by replacement of the endogenous 3′ UTR in the NF54^Cas9+T7 Polymerase^ strain with the anhydrotetracycline-regulated aptamer sequence and the TetR-DOZI cassette. (B) Anti-myc Western blot on the ATG8-TetR lysate. The myc-BirA-expressing strain was used as a positive control for the anti-myc antibody. An anti-ATG8 blot is shown for comparison. Aldolase, loading control. Asterisk indicates unspecific bands. Download FIG S1, PDF file, 0.5 MB. Copyright © 2018 Walczak et al. 2018 Walczak et al. This content is distributed under the terms of the Creative Commons Attribution 4.0 International license.

The growth inhibition observed in ATG8-deficient parasites may specifically be due to its function in the apicoplast or a result of other functions. To distinguish between essential apicoplast and nonapicoplast ATG8 functions, we determined the growth of ATG8-deficient parasites in medium supplemented with isopentenyl pyrophosphate (IPP). We previously showed that IPP is the only essential product of the apicoplast in blood-stage *Plasmodium*. As such, any disruption of the apicoplast, including complete loss of the organelle, can be rescued by the addition of IPP (25). IPP fully rescued the growth defect of ATG8-deficient parasites, demonstrating that the only essential function of ATG8 is specific to the apicoplast (Fig. 1D). *Pf*ATG8 may have other functions in blood-stage *Plasmodium* that are not essential but are important for parasite growth fitness, which was not assessed in this study.

### ATG8 depletion leads to terminal loss of the apicoplast in growth-rescued parasites.

**FIG 2:**
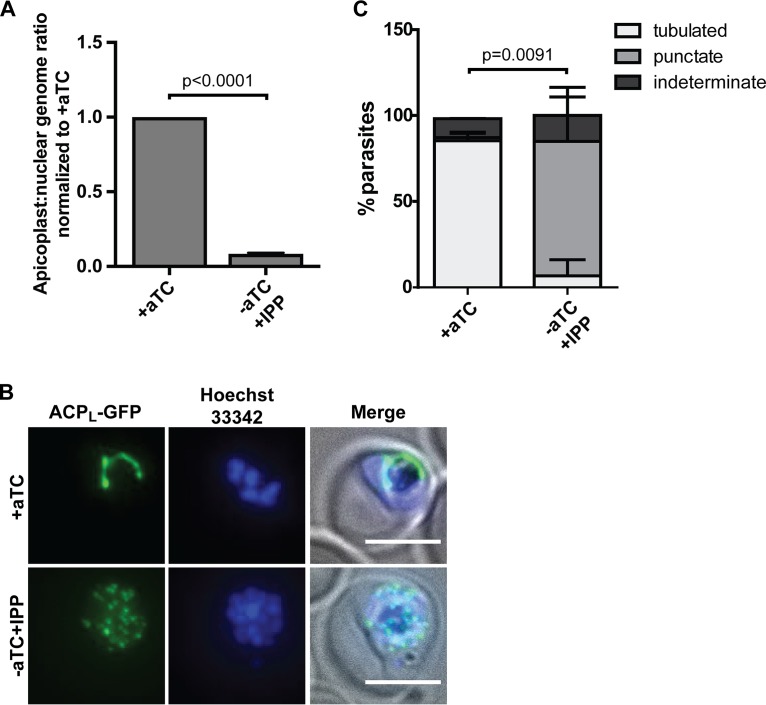
ATG8 depletion leads to terminal apicoplast loss. (A) Apicoplast:nuclear genome ratio in ATG8-deficient, IPP-rescued parasites (grown for 4 cycles without aTC) measured by quantitative PCR. The ratios were normalized to ATG8^+^ culture (i.e., grown in the presence of aTC). Mean ± standard deviation for 2 biological replicates is shown. The *P* value was determined by unpaired two-tailed *t* test. (B) Representative microscopy images showing localization of apicoplast-targeted GFP (ACP_L_-GFP), in schizont-stage ATG8^+^ or ATG8-deficient/IPP-rescued parasites depleted of ATG8 for 2 replication cycles. Bar, 5 µm. (C) Quantification of parasites with the indicated apicoplast morphology in ATG8^+^ or ATG8-deficient/IPP-rescued parasites as shown in panel B. Mean ± standard deviation of 2 independent experiments is shown. The *P* value was determined by unpaired two-tailed *t* test for the “tubulated” category. Data for separate replicates are shown in Fig. S2.

Each parasite contains a single apicoplast which must be replicated and inherited during cell division. To determine whether *Pf*ATG8 is required for apicoplast biogenesis during parasite replication, we assessed the presence of the apicoplast in ATG8-deficient, IPP-rescued parasites after ≥2 replication cycles when the terminal effects of ATG8 deficiency would be apparent (25). In the first assay, we measured the copy number of the apicoplast genome compared to the nuclear genome and detected a 10-fold decrease in the apicoplast/nuclear genome ratio (Fig. 2A). In a second assay, we determined the localization of an apicoplast-targeted green fluorescent protein (ACP_L_-GFP). In schizont-stage parasites expressing ATG8, ACP_L_-GFP localized to tubular structures which resemble the distinctive branched apicoplast in this stage. In contrast, in ATG8-deficient, IPP-rescued parasites ACP_L_-GFP mislocalized to cytosolic puncta, similar to what has previously been observed in parasites in which apicoplast loss has been induced by treatment with apicoplast transcription and translation inhibitors like chloramphenicol (Fig. 2B and C and S2) (25, 26). Altogether, ATG8 depletion results in terminal loss of the apicoplast, likely due to a failure to replicate and inherit new apicoplasts during parasite replication.

10.1128/mBio.02021-17.2 FIG S2  (A) Separate biological replicates of the experiment shown in Fig. 2B and C. Nine and 15 parasites for replicate 1 and 55 and 59 parasites for replicate 2 were counted for +aTC and −aTC conditions, respectively. Download FIG S2, PDF file, 0.2 MB. Copyright © 2018 Walczak et al. 2018 Walczak et al. This content is distributed under the terms of the Creative Commons Attribution 4.0 International license.

### ATG8 knockdown does not affect protein and lipid import to the apicoplast.

We noted that parasite growth was initially unaffected by ATG8 knockdown but then decreased drastically in the subsequent replication cycle. As seen in Fig. 1C and D, despite substantial ATG8 depletion upon aTC removal, ATG8-deficient parasites reinvaded new host cells, efficiently achieving similar parasitemia as ATG8^+^ parasites in cycle 1. However, in the subsequent reinvasion (cycle 2), the parasitemia was 26% of the control. To determine whether ATG8 depletion caused defects in apicoplast biogenesis in cycle 1 that preceded the block in parasite replication in cycle 2, we monitored key events in apicoplast biogenesis in the first cycle of ATG8 knockdown (see Fig. 5A).

**FIG 3:**
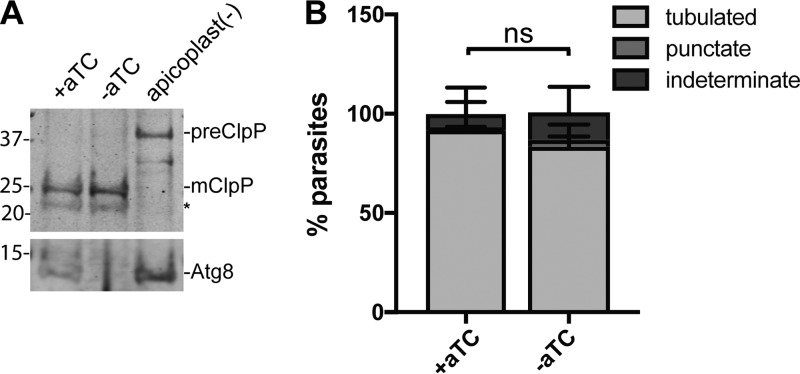
Apicoplast protein import and membrane expansion are not affected in the first cycle of ATG8 knockdown. (A) Processing of a luminal apicoplast protein, ClpP, in ATG8^+^ or ATG8-deficient parasites approximately 24 h after aTC removal. Apicoplast-minus parasites generated by chloramphenicol treatment and IPP rescue over 4 replication cycles (25), which possess only precursor ClpP, are shown for reference. preClpP, full-length (precursor) form of ClpP, 43 kDa; mClpP, mature (apicoplast-luminal) ClpP, 25 kDa. The asterisk indicates a nonspecific band. ATG8 expression in the corresponding time points is shown for reference. Numbers on the left are molecular masses in kilodaltons. (B) Quantification of parasites with the indicated apicoplast morphology during the first cycle of ATG8 knockdown, 32 h after aTC removal. Apicoplast was visualized using the luminal apicoplast marker ACP_L_-GFP. Mean ± standard deviation of 2 independent experiments is shown. The *P* value was determined by unpaired two-tailed *t* test for the “tubulated” category. Representative images and data for separate replicates are shown in Fig. S3. ns, not significant.

The first distinctive change associated with apicoplast biogenesis is growth and formation of a branched apicoplast (27, 28), which is likely dependent on protein and lipid import (see Fig. 5A). Apicoplast-targeted proteins possess an N-terminal transit peptide sequence which targets them to the apicoplast and is removed upon import into the apicoplast (29). To assess apicoplast protein import in ATG8-deficient parasites, we monitored the processing of an imported protein, ClpP, from a 43-kDa full-length protein containing an intact transit peptide (as observed in chloramphenicol-induced apicoplast loss) to a 25-kDa mature form (30). We observed no defect in ClpP processing in trophozoite parasites ~24 h after ATG8 knockdown (Fig. 3A). Furthermore, apicoplast-targeted ACP_L_-GFP localized to a branched tubular structure similar to those in ATG8^+^ parasites in schizont parasites ~32 h after ATG8 knockdown, indicating that lipid import contributing to this extensive membrane expansion was also unaffected (Fig. 3B and S3). Our data suggest that ATG8 expression is not immediately required for apicoplast protein import or membrane expansion.

10.1128/mBio.02021-17.3 FIG S3  (A) Representative live images of ACP_L_-GFP-expressing schizont-stage parasites in the first cycle of ATG8 knockdown, i.e., approximately 32 h after aTC removal. Bar, 5 µm. (B) Separate biological replicates of the experiment shown in Fig. 3B. Thirteen and 43 parasites for replicate 1 and 34 and 55 parasites for replicate 2 were counted for +aTC and −aTC conditions, respectively. Download FIG S3, PDF file, 0.4 MB. Copyright © 2018 Walczak et al. 2018 Walczak et al. This content is distributed under the terms of the Creative Commons Attribution 4.0 International license.

### ATG8 knockdown results in a late block in apicoplast inheritance.

The final events in apicoplast biogenesis are division of the branched apicoplast to form multiple plastids and segregation of a single apicoplast into each forming daughter parasite (merozoite [see Fig. 5A]). These events required for organelle inheritance have not been directly observed. Instead, we assessed apicoplast inheritance upon ATG8 knockdown by detecting the presence of the apicoplast genome and apicoplast-targeted ACP_L_-GFP in newly reinvaded ATG8-deficient parasites (48 h after aTC removal and ~12 h post invasion) after the first cycle of ATG8 knockdown. As noted, ATG8-deficient parasites reinvaded to similar parasitemia levels as ATG8^+^ parasites in this first reinvasion (Fig. 1D).

**FIG 4:**
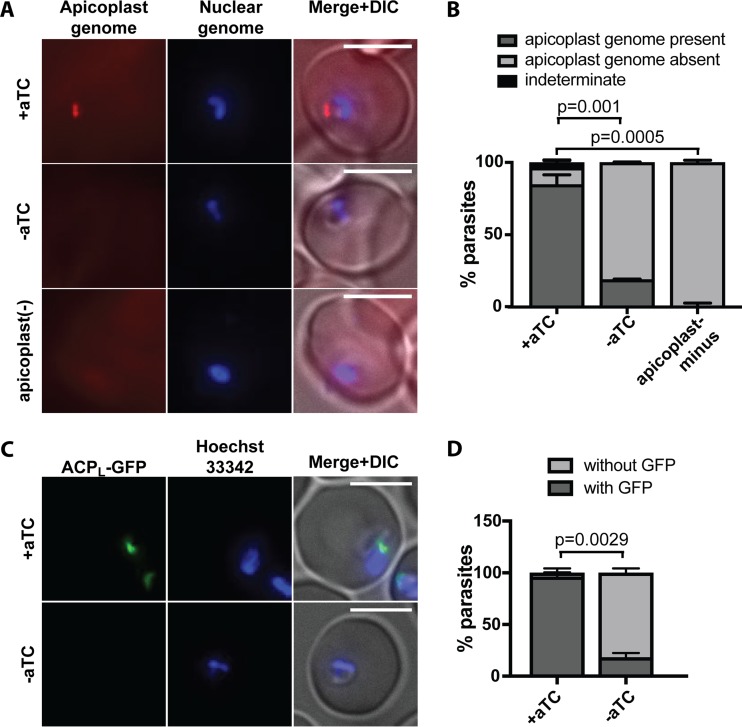
ATG8 knockdown leads to defects in apicoplast inheritance**.** (A) Representative images of apicoplast FISH detecting the apicoplast genome in ring-stage ATG8^+^ and ATG8-deficient parasites 48 h after aTC removal, i.e., after first reinvasion (top and middle rows, respectively). Apicoplast-minus parasites, generated by 4 cycles of chloramphenicol treatment and IPP rescue, are shown as a negative control for FISH staining (bottom row). Bars, 5 µm. (B) Quantification of parasites with or without apicoplast genome grown under indicated conditions. Mean ± standard deviation for 2 independent experiments as in panel A is shown. The *P* values, determined by one-way analysis of variance, are shown for the “apicoplast genome present” category. Data for separate replicates are in Fig. S4. (C) Representative images of ring-stage ATG8^+^ or ATG8-deficient parasites expressing ACP_L_-GFP 48 h after aTC removal, i.e., after first reinvasion. Bars, 5 µm. (D) Quantification of parasites with or without a discrete GFP-labeled structure as shown in panel C grown under indicated conditions. Mean ± standard deviation for 2 independent experiments is shown. The *P* value was determined by unpaired two-tailed *t* test for the “with GFP” category. Data for separate replicates are shown in Fig. S4. DIC, differential interference contrast.

To detect the single-copy apicoplast genome with single-cell resolution, we developed a fluorescence *in situ* hybridization (FISH) protocol using an Oligopaint library of 477 FISH probes covering >60% of the 35-kb genome (apicoplast FISH) (31, 32). As expected, a majority of ATG8^+^ parasites (85%) had a single fluorescent punctum corresponding to the apicoplast genome (Fig. 4A and B and S4). This punctum was absent from negative-control parasites in which apicoplast loss had been induced by chloramphenicol treatment, demonstrating that apicoplast FISH was specific (Fig. 4A and B and S4) (25). In contrast to ATG8^+^ parasites, only 19% of reinvaded ATG8-deficient parasites contained an apicoplast genome after the first cycle of ATG8 knockdown (Fig. 4A and B and S4). Since the experiments were performed on a nonclonal population, the small percentage of apicoplast FISH-positive parasites in the ATG8-deficient pool was likely due to incomplete ATG8 knockdown or unmodified wild-type parasites.

10.1128/mBio.02021-17.4 FIG S4  (A) Separate biological replicates of the experiment shown in Fig. 4A and B. A minimum of 110 parasites were counted per condition per single experiment. (B) Separate biological replicates of the experiment shown in Fig. 4C and D. A minimum of 68 parasites were counted per condition per single experiment. Download FIG S4, PDF file, 0.5 MB. Copyright © 2018 Walczak et al. 2018 Walczak et al. This content is distributed under the terms of the Creative Commons Attribution 4.0 International license.

Similarly, we detected ACP_L_-GFP in individual ring-stage parasites by fluorescence microscopy (Fig. 4C and D and S4). Consistent with apicoplast FISH results, 96% of ATG8^+^ parasites had a punctate or elongated ACP_L_-GFP signal, while only 18% of ATG8-deficient parasites contained detectable ACP_L_-GFP. The presence of the apicoplast genome and apicoplast-localized protein in these early ring-stage parasites should reflect inheritance of the organelle, rather than DNA or protein synthesis, since neither genome replication nor GFP expression is active in this stage. Therefore, the decrease in apicoplast FISH- and ACP_L_-GFP-labeled structures in the progeny of ATG8-deficient parasites suggests that apicoplast inheritance was disrupted.

## DISCUSSION

Our findings demonstrate that *Pf*ATG8 has a novel, essential function in apicoplast biogenesis which is conserved among apicomplexan parasites. *Pf*ATG8 is essential in blood-stage *Plasmodium* and required for apicoplast biogenesis. Consistent with this, other ATG proteins required for ATG8 lipidation are also important in blood-stage *Plasmodium*: *Pf*ATG7 was shown to be essential for parasite replication (17). A recent study (published while this paper was under revision) showed that *Pf*ATG18 is essential in the blood stage and required for apicoplast biogenesis (33). The essentiality of apicomplexan ATG8 homologs contrasts with yeast and mammalian ATG8s, which are not strictly required for cell growth and proliferation under nutrient-replete conditions (14, 15). Moreover, though ATG8 has been proposed to have diverse functions in *Plasmodium* parasites from starvation-induced autophagy to stage-specific organelle turnover to intracellular vesicle trafficking (6–8), we showed that only its role in apicoplast biogenesis is essential for blood-stage *Plasmodium* replication. *Tg*ATG8’s essentiality was also attributed to its apicoplast function, since neither autophagosome biogenesis by ATG9 nor proteolysis in the lysosomal compartment is essential in replicating tachyzoites (34–36).

The unique function of *Pf*ATG8 may be leveraged for antimalarial drug development. Since autophagy has important roles in mammalian physiology and development, specificity for disruption of *Pf*ATG8 and its conjugation will be imperative. One strategy may be to identify druggable targets downstream of *Pf*ATG8 that specifically affect apicoplast biogenesis (15, 37), though it is unclear whether direct inhibition of ATG8 function (as opposed to interfering with its expression) will result in the delayed growth inhibition observed in our ATG8-knockdown strain. Finally, we determined essential *Pf*ATG8 functions for blood-stage *P. falciparum* growth using an *in vitro* culture system; it is possible that ATG8 has other essential functions under *in vivo* conditions and/or in other life stages.

*Pf*ATG8 has a novel function in apicoplast biogenesis. Because ATG8 homologs in model eukaryotes have not previously been implicated in biogenesis of mitochondria or primary chloroplasts, this function likely evolved as a result of secondary endosymbiosis in this parasite lineage. The repurposing of a conserved eukaryotic protein for the biogenesis of a secondary plastid is at first surprising. However, ATG8-conjugated membranes of the apicoplast and autophagosomes both have their origins in the endomembrane system. The endoplasmic reticulum (ER) and ER-associated membranes are the main membrane source of autophagosomes (38–43). Meanwhile, ATG8 is conjugated to the outermost of 4 apicoplast membranes (16), which derives from the host endomembrane during secondary endosymbiosis (1). Indeed, apicoplast biology has numerous tantalizing connections to ER biology. Protein import into the apicoplast requires that nucleus-encoded proteins traffic to the ER en route to the apicoplast (29). A translocon related to the ER-associated protein degradation (ERAD) system localizes to the apicoplast and may be involved in protein import, another example of an ER-associated membrane function that has been repurposed for apicoplast function (44–47). Finally, in some free-living protists, the secondary plastid is located within the ER with the outermost membrane of the plastid contiguous with the ER (48, 49). The endomembrane origin of the outer membrane may explain the novel function of ATG8 in apicoplast biogenesis.

What is the function of ATG8 on this outermost membrane? Mammalian and yeast ATG8 homologs have two unique properties that contribute to their diverse autophagy-related and autophagy-independent functions. First, they stimulate membrane tethering, hemifusion, and fusion, important for their role in autophagosome formation (50, 51). Based on this membrane fusion activity, *Pf*ATG8 was proposed to promote membrane expansion of a growing apicoplast and/or fusion of vesicles containing nucleus-encoded proteins with the apicoplast (6–8, 10, 12, 52). However, in ATG8-deficient *P. falciparum*, we did not observe any defect in either membrane expansion (assayed by formation of a branched intermediate) or protein import (assayed by ClpP transit peptide processing) prior to the block in parasite replication. We therefore consider these putative functions less likely.

**FIG 5:**
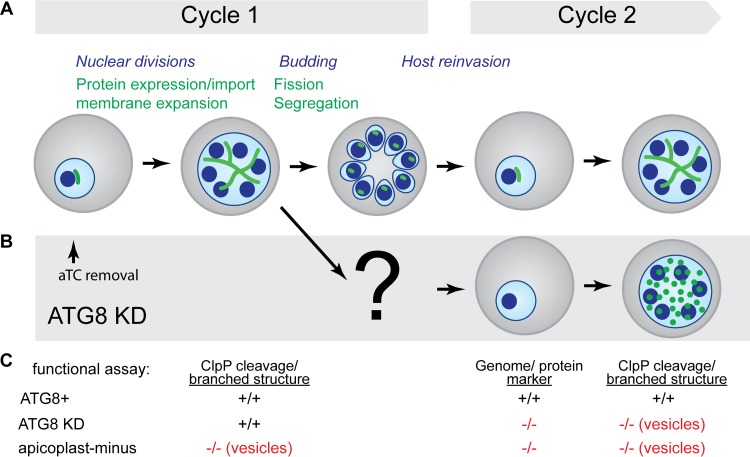
Schematic summarizing apicoplast defects detected in ATG8-deficient parasites. (A) Time course of cellular events during blood-stage development of *Plasmodium* parasites (the apicoplast is shown in green, the parasite nuclei and cell membrane are shown in blue, and the host erythrocyte is shown in gray). Steps in apicoplast development are indicated in green font, and those in parasite replication are indicated in italic blue font. (B) Apicoplast biogenesis defects observed upon ATG8 knockdown (KD) are shown. The timing of aTC removal to start ATG8 knockdown in ring-stage parasites is indicated (arrow). (C) Summary of functional assays performed in this study to assess the effects of ATG8 knockdown on specific steps in apicoplast development listed in panel A. The results of these functional assays are compared in ATG8^+^, ATG8 knockdown, and apicoplast-minus parasites, with defects highlighted in red font.

Second, ubiquitin-like Atg8 proteins are versatile protein scaffolds for membrane complexes, interacting with a variety of effector proteins, including cargo receptors, soluble *N*-ethylmaleimide-sensitive factor (NSF) attachment protein receptors (SNAREs), NSF, Rab GTPase activating proteins (GAPs), and microtubules (53–59). In ATG8-deficient parasites, the earliest defect that we observed upon ATG8 knockdown was in apicoplast inheritance, resulting in progeny after the first reinvasion that lack a functional apicoplast and fail to replicate (Fig. 5B and C). We propose that *Pf*ATG8 is required to resolve the branched intermediate into individual apicoplasts (fission) and/or facilitate the distribution of a single apicoplast into each budding daughter parasite (segregation). Failure of either fission or segregation could lead to exclusion of the apicoplast from daughter parasites and its accumulation in the residual body, the parent cell remnants following division of merozoites (16). Alternatively, improper segregation could result in uneven distribution of apicoplasts, with some merozoites inheriting several apicoplasts and some inheriting none. Although a small percentage of ATG8-deficient parasites contained distinct apicoplast FISH- or GFP-positive structures, we did not observe any apparent difference in their size or fluorescence intensity compared to those in ATG8^+^ parasites. Therefore, we surmise that (i) the minority of ATG8-deficient parasites containing detectable apicoplast markers was due to incomplete ATG8 knockdown and (ii) in the majority of ATG8-deficient parasites, the apicoplast was lost in the residual body.

Are there known ATG8 effectors that provide a model for these functions? To our knowledge, interaction of ATG8 homologs with normal-topology membrane fission machinery, such as dynamins, has not been reported (60). Mammalian ATG8 homologs LC3 and gamma-aminobutyric acid (GABA) type A receptor-associated protein (GABARAP) have been shown to interact directly and indirectly with centrosomal proteins, albeit not conserved in apicomplexans (61–63). In *T. gondii* and another apicomplexan, *Sarcocystis neurona*, dividing apicoplasts are associated with centrosomes, which may serve as a counting mechanism to ensure inheritance of a single apicoplast by each daughter parasite (64, 65). Notably, the association is independent of the mitotic spindle and lost upon knockdown of ATG8 in *T. gondii*, suggesting that ATG8 may mediate this interaction with centrosomal proteins (16, 65). Though *Plasmodium* lacks centrioles and instead contains “centrosome-like” structures, apicoplast-bound ATG8 may interact with these structures in *Plasmodium* as well (66, 67). Finally, LC3 and GABARAPs also interact with microtubules and may be required for the transport of autophagosomes and GABA receptor-containing vesicles, respectively (56, 58, 59, 68). By analogy, *Pf*ATG8 may interact with microtubules to position the apicoplast during parasite division. Indeed, *Pf*ATG8 may interact with multiple effectors at the apicoplast membrane, as it does on autophagosomes, to ensure organelle inheritance. Identifying these effectors will be a challenging but critical next step.

ATG8’s function in apicoplast biogenesis is required in different life stages of *Plasmodium* spp. and conserved among related apicomplexan parasites. Our results in blood-stage *Plasmodium* corroborate findings in liver-stage *Plasmodium* and *T. gondii* tachyzoites that also showed a role in apicoplast biogenesis (8, 10, 12, 16). In fact, the apicoplast function of apicomplexan ATG8 is the most consistently observed. Even autophagy, which is the “ancestral” function of ATG8, is not clearly preserved in *Plasmodium* parasites. It will be interesting to determine whether ATG8’s role in apicoplast biogenesis is a specific adaptation of apicomplexan parasites or is also found in free-living relatives that possess a secondary plastid of the same origin, such as *Chromera*. Overall, the evolution of this new protein function for a key endosymbiotic event from an ancient template is intriguing (69).

## MATERIALS AND METHODS

### Culture and transfection conditions.

*Plasmodium falciparum* parasites were grown in human erythrocytes (Research Blood Components, Boston, MA/Stanford Blood Center, Stanford, CA) at 2% hematocrit under 5% O_2_ and 5% CO_2_, at 37°C in RPMI 1640 medium supplemented with 5 g/liter AlbuMAX II (Gibco), 2 g/liter NaHCO_3_ (Fisher), 25 mM HEPES (pH 7.4) (Sigma), 0.1 mM hypoxanthine (Sigma), and 50 mg/liter gentamicin (Gold Biotechnology) (further referred to as culture medium). For transfections, 50 µg plasmid DNA was used per 200 µl packed red blood cells (RBCs), adjusted to 50% hematocrit, and electroporated as previously described (22). Parasites were selected with a combination of 2.5 mg/liter blasticidin S (RPI Research Products) and 2.5 nM WR99210 (ATG8 TetR strain) or 2.5 mg/liter blasticidin S and 500 µg/ml G418 sulfate (Corning) (ACP_L_-GFP-expressing ATG8-TetR strain) beginning 4 days after transfection.

### Cloning and strain generation.

All primers used for this study are listed in Table S1 in the supplemental material. *P. falciparum* NF54^attB^ parasites (kindly provided by David Fidock) engineered to continuously express Cas9 and T7 RNA polymerase (NF54^Cas9+T7 Polymerase^) (70) were used as a parental strain for deriving ATG8 conditional knockdown parasites.

10.1128/mBio.02021-17.5 TABLE S1  List of primers used in this study. Download TABLE S1, PDF file, 0.05 MB. Copyright © 2018 Walczak et al. 2018 Walczak et al. This content is distributed under the terms of the Creative Commons Attribution 4.0 International license.

The construct for inducible ATG8 expression, pSN053-ATG8, was created by cloning left and right homology arms and guide RNA into a pJazz system-based vector, pSN053. The pSN053 vector contains (i) a C-terminal myc tag followed by a 10× aptamer array for anhydrotetracycline-dependent regulation of translation; (ii) a TetR-DOZI cassette containing the *Renilla* luciferase (RLuc) gene for monitoring transfection, the blasticidin resistance gene for selection, and a TetR-DOZI repressor, with PfHrp2 3′ and PfHsp86 5′ regulatory sequences, in head-to-head orientation with the modified gene; and (iii) a guide RNA expression cassette with T7 promoter and T7 terminator. The left homology arm was amplified from genomic DNA with primers SMG413 and SMG425 and inserted into the FseI-AsiSI site in frame with the myc tag. The right homology arm was amplified from genomic DNA with the primers SMG411 and SMG412 and inserted into the I-SceI and I-CeuI sites downstream of the TetR-DOZI cassette. The guide RNA was generated by Klenow reaction from oligonucleotides SMG419 and SMG420 and inserted into the AflII site. All ligation steps were performed using Gibson assembly. The resulting plasmid was transfected into the NF54^Cas9+T7 Polymerase^ strain as described above, and transformants were selected with 2.5 µg/liter blasticidin S and 2.5 nM WR99210. Culture was maintained in 0.5 µM anhydrotetracycline (aTC) (Sigma) unless stated otherwise. Transgene integration (5′ junction) was confirmed by PCR using primers SMG454 and SMG493. We were not able to amplify a product on the 3′ junction. This strain is referred to as the ATG8-TetR strain.

To introduce a fluorescent apicoplast marker, GFP with an apicoplast targeting leader sequence, ACP_L_-GFP was amplified from pRL2-ACP_L_-GFP using primers mawa059 and mawa060 and cloned into AvrII-SacII restriction sites of a pYC110F plasmid (71) using InFusion (Clontech). The plasmid was transfected into the ATG8-TetR strain, and transformants were selected with 2.5 mg/liter blasticidin S and 500 mg/liter G418 sulfate. Cultures were maintained in 0.5 µM aTC and 500 mg/liter G418 sulfate.

### ATG8 knockdown experiments.

Ring-stage parasites at 5 to 10% parasitemia were washed twice in the culture medium to remove aTC and resuspended in the culture medium, and the hematocrit was adjusted to 2%. Parasites were divided into 3 cultures grown in the culture medium supplemented with 0.5 µM aTC, without aTC, or without aTC but with 200 µM IPP (isoprenoids) for 4 replication cycles. At the schizont stage of each cycle, cultures were diluted 5-fold into fresh culture medium with red blood cells at 2% hematocrit and aTC or IPP was added as required. Aliquots of culture for Western blotting, quantitative PCR, and flow cytometry were collected at the ring and/or schizont stage of each cycle before diluting the cultures.

### Flow cytometry.

Parasite cultures or uninfected RBCs at 2% hematocrit were fixed with 1% paraformaldehyde (Electron Microscopy Solutions) in phosphate-buffered saline (PBS) for 4 h at room temperature (RT) or overnight at 4°C. Nuclei were stained with 50 nM YOYO-1 (Life Technologies) for a minimum of 1 h at room temperature. Parasites were analyzed on the BD Accuri C6 flow cytometer. Measurements were done in technical triplicates.

### Western blotting.

Parasites were lysed with 1% saponin for 5 min on ice. Parasite pellets were washed twice with ice-cold PBS and resuspended in 20 µl 1× lithium dodecyl sulfate (LDS) buffer (Life Technologies) per 1 ml culture at 2% hematocrit and 5% parasitemia. Equal parasite numbers were loaded per lane. After separation on Bis-Tris Novex gels (Invitrogen), proteins were transferred to a nitrocellulose membrane, blocked with a buffer containing 0.1% casein (Hammarsten; Affymetrix) and 0.2× PBS, and incubated with the corresponding antibodies diluted in 50% blocking buffer–50% Tris-buffered saline–Tween (TBST). Primary antibodies were used overnight at a 1:1,000 dilution, except antialdolase, which was used at 1:10,000, and anti-GFP, used at 1:20,000. Secondary antibodies were used at a 1:10,000 dilution for 1 h at room temperature. Blots were visualized using the LiCor double-color detection system and converted to grayscale images for the purpose of this publication. The following antibodies were used: anti-ATG8, from Josman LLC (see below); anti-aldolase (Abcam catalog no. ab207494); anti-ClpP, a gift from W. Houry (30); and anti-GFP (Clontech catalog no. 632381). Fluorophore-conjugated IRDye secondary antibodies were purchased from Fisher (LiCor).

### Quantitative PCR.

An 0.5-ml amount of culture was lysed with 1% saponin and washed twice with PBS. DNA was purified using the DNeasy Blood and Tissue kit (Qiagen). PCR mixtures were prepared using LightCycler 480 SYBR green I master mix (Roche) according to the manufacturer’s instructions and run in triplicates on the Applied Biosystems 7900HT cycler. Primers TufA fwd and TufA rev were used for the apicoplast target, and Cht1 fwd and Cht1 rev were used for the nuclear target. Cycling conditions were 95°C for 10 min; 35 cycles of 95°C for 30 s, 56°C for 30 s, and 65°C for 90 s; 65°C for 5 min; and a melting curve of 65°C to 95°C. Data were analyzed using a threshold cycle (ΔΔ*C*_*T*_) method as previously described (72).

### Fluorescence microscopy.

Live or fixed parasites were stained with 2 µg/ml Hoechst 33342 stain for 15 min at room temperature to visualize nuclei. Images were acquired using the Olympus IX70 microscope equipped with a DeltaVision Core system, a 100× 1.4-numerical-aperture (NA) Olympus lens, a Sedat Quad filter set (Semrock), and a CoolSnap HQ charge-coupled device (CCD) camera (Photometrics) controlled via softWoRx 4.1.0 software. Images were analyzed using ImageJ.

### Fluorescence *in situ* hybridization.

Oligopaint FISH probe library MyTag was purchased from MYcroarray (see Table S2). The library consisted of 477 high-stringency Atto-550-conjugated probes with an overall probe density of 13.9 probes per kb of the apicoplast genome. The probes were resuspended to 10 pmol/µl in ultrapure water (stock solution).

10.1128/mBio.02021-17.6 TABLE S2  Sequences of the Oligopaint library probes used for apicoplast FISH in this study (see separate Excel file). Download TABLE S2, XLSX file, 0.1 MB. Copyright © 2018 Walczak et al. 2018 Walczak et al. This content is distributed under the terms of the Creative Commons Attribution 4.0 International license.

The fluorescence *in situ* hybridization protocol was adapted from reference 32. Infected red blood cells (iRBCs) were washed twice with PBS and fixed with 10 volumes of the fixation solution (4% paraformaldehyde [Electron Microscopy Solutions catalog no. 50-980-487], 0.02% glutaraldehyde [Sigma catalog no. G6257] in PBS) for 1 h at 37°C. Fixed iRBCs were washed twice with PBS and permeabilized with 1% Triton X-100 in PBS for 10 min at room temperature, followed by 3 washes in PBS. Next, parasites were resuspended in the hybridization solution (50% [vol/vol] formamide [Sigma], 10% dextran sulfate [Millipore], 2× SSPE [1× SSPE is 0.18 M NaCl, 10 mM NaH_2_PO_4_, and 1 mM EDTA {pH 7.7}] [Sigma], 250 mg/ml salmon sperm DNA [Sigma]) to approximately 20% hematocrit and incubated for 30 min at 37°C. MyTag probes were resuspended in the hybridization solution to a final concentration of 1 pmol/µl, denatured for 5 min at 100°C, and cooled on ice. Fifty microliters resuspended parasites was added to 20 µl of hybridization solution with or without probes and incubated for 30 min at 80°C followed by a minimum of 16 h of incubation at 37°C. Next, iRBCs were subjected to the following washes: 30 min at 37°C in 50% (vol/vol) formamide, 2× SSC (1× SSC is 0.15 M NaCl plus 0.015 M sodium citrate) (Sigma); 10 min at 50°C in 1× SSC; 10 min at 50°C in 2× SSC; 10 min at 50°C in 4× SSC; and 10 min at 50°C in PBS. Cells were resuspended in 50 µl PBS, stained with 2 µg/ml Hoechst 33342, and imaged.

### ATG8 purification and anti-ATG8 antibody production.

Hexahistidine-tagged ATG8 was expressed in Rosetta DE3 with a rare codon plasmid from pRSF-1b-His-ATG8 (73, 74). Bacterial cultures were grown in terrific broth (TB) medium supplemented with 50 mg/liter kanamycin and 34 mg/liter chloramphenicol. At an optical density at 600 nm (OD_600_) of 3, IPTG (isopropyl-β-d-thiogalactopyranoside) was added to the final concentration of 300 µM to induce ATG8 expression, and cultures were further grown at 20°C for 16 h. Bacteria were harvested by 20 min of centrifugation at 800 × *g*, and bacterial pellets were resuspended in the buffer containing 50 mM HEPES (pH 8.0), 500 mM NaCl, 1 mM MgCl_2_, 10% glycerol, and 2× complete protease inhibitors (Pierce). Cells were lysed by a series of freeze-thaw cycles followed by passing them 3 times through the Avestin EmulsiFlex emulsifier. Cell debris was removed by 30 min of centrifugation at 30,000 × *g*. Clarified lysate was added to Talon resin (Clontech) and incubated for 1 h at 4°C. Beads were washed with the wash buffer containing 50 mM HEPES (pH 8.0), 150 mM NaCl, 10 mM imidazole (pH 8.0), and 10% glycerol. Protein was eluted with the wash buffer supplemented with 300 mM imidazole (pH 8.0), dialyzed against the wash buffer lacking imidazole, aliquoted, and stored at −80°C. Anti-ATG8 antibodies were raised in a rat and a guinea pig at Josman LLC. Josman is a research facility licensed through the USDA, number 93-R-0260, and has a PHS Assurance from the OLAW of the NIH, number A3404-01.
