# Supplementary material for: ATG8 Is Essential Specifically for an Autophagy-Independent Function in Apicoplast Biogenesis in Blood-Stage Malaria Parasites
Source: mBio. 2018 Jan 2;9(1):e02021-17. doi: 10.1128/mBio.02021-17 (PMC5750400; doi:10.1128/mBio.02021-17)
Supplement: FIG S1 [file mbo001183655sf1.pdf]

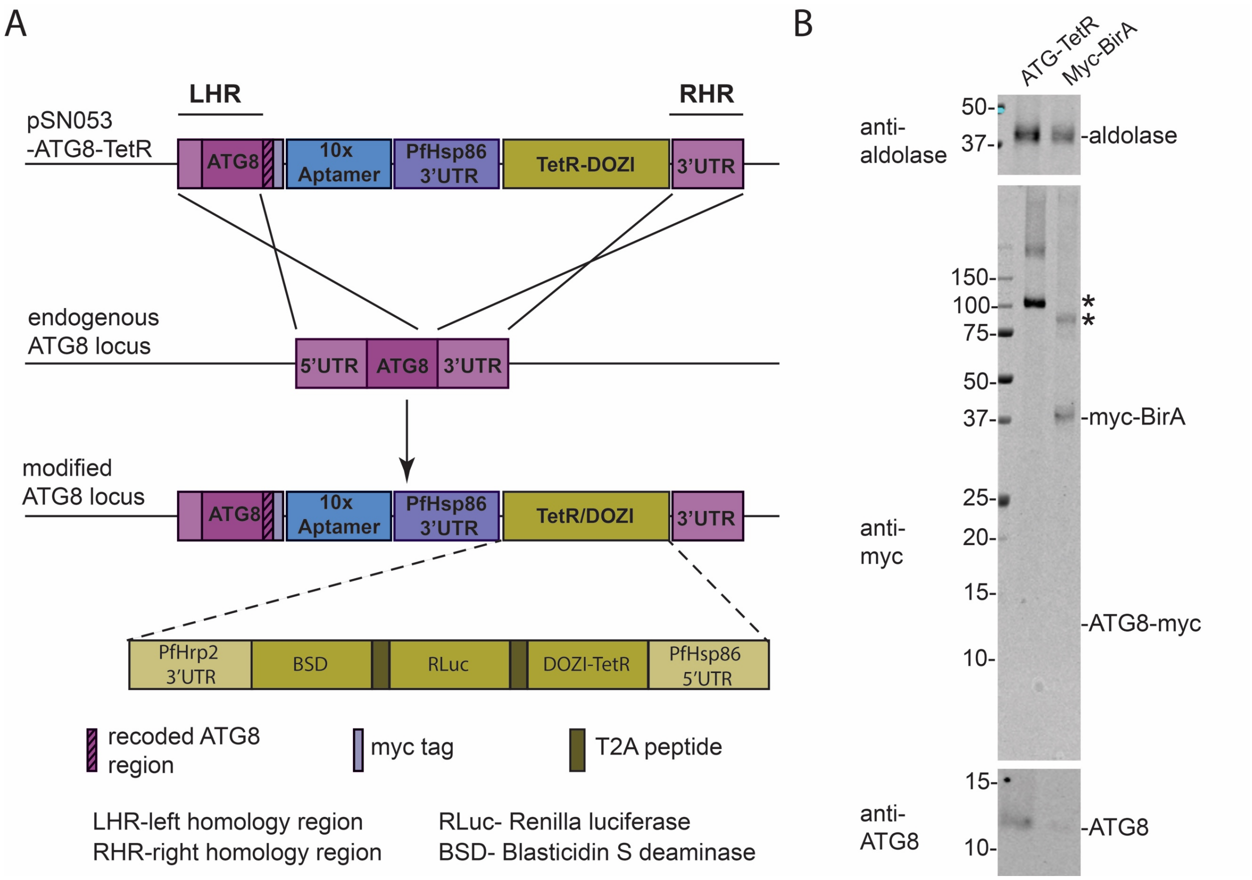

Supplementary Figure S1

(A) Conditional ATG8 knockdown line (ATG8-TetR) generated by replacement of the endogenous 3' UTR in the NF54$^{Cas9+T7\ Polymerase}$ strain with the anhydrotetracycline-regulated aptamer sequence and the TetR-DOZI cassette. (B) Anti-myc western blot on the ATG8-TetR lysate. Myc-BirA-expressing strain was used as a positive control for the anti-myc antibody. Anti-ATG8 blot is shown for comparison. Aldolase, loading control. Asterisk indicates unspecific bands.
