# Supplementary material for: ATG8 Is Essential Specifically for an Autophagy-Independent Function in Apicoplast Biogenesis in Blood-Stage Malaria Parasites
Source: mBio. 2018 Jan 2;9(1):e02021-17. doi: 10.1128/mBio.02021-17 (PMC5750400; doi:10.1128/mBio.02021-17)
Supplement: FIG S2 [file mbo001183655sf2.pdf]

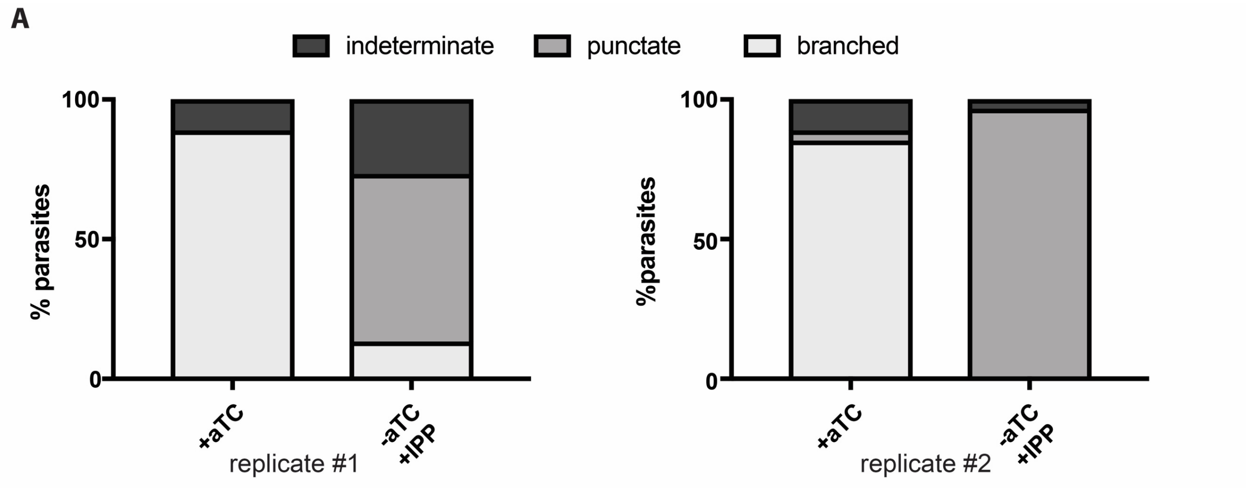

Supplementary Figure S2

(A) Separate biological replicates of the experiment shown in Figure 2B-C. Nine and 15 parasites for replicate #1, and 55 and 59 parasites for replicate 2 were counted for +aTC and –aTC condition, respectively.
