# Supplementary material for: ATG8 Is Essential Specifically for an Autophagy-Independent Function in Apicoplast Biogenesis in Blood-Stage Malaria Parasites
Source: mBio. 2018 Jan 2;9(1):e02021-17. doi: 10.1128/mBio.02021-17 (PMC5750400; doi:10.1128/mBio.02021-17)
Supplement: FIG S3 [file mbo001183655sf3.pdf]

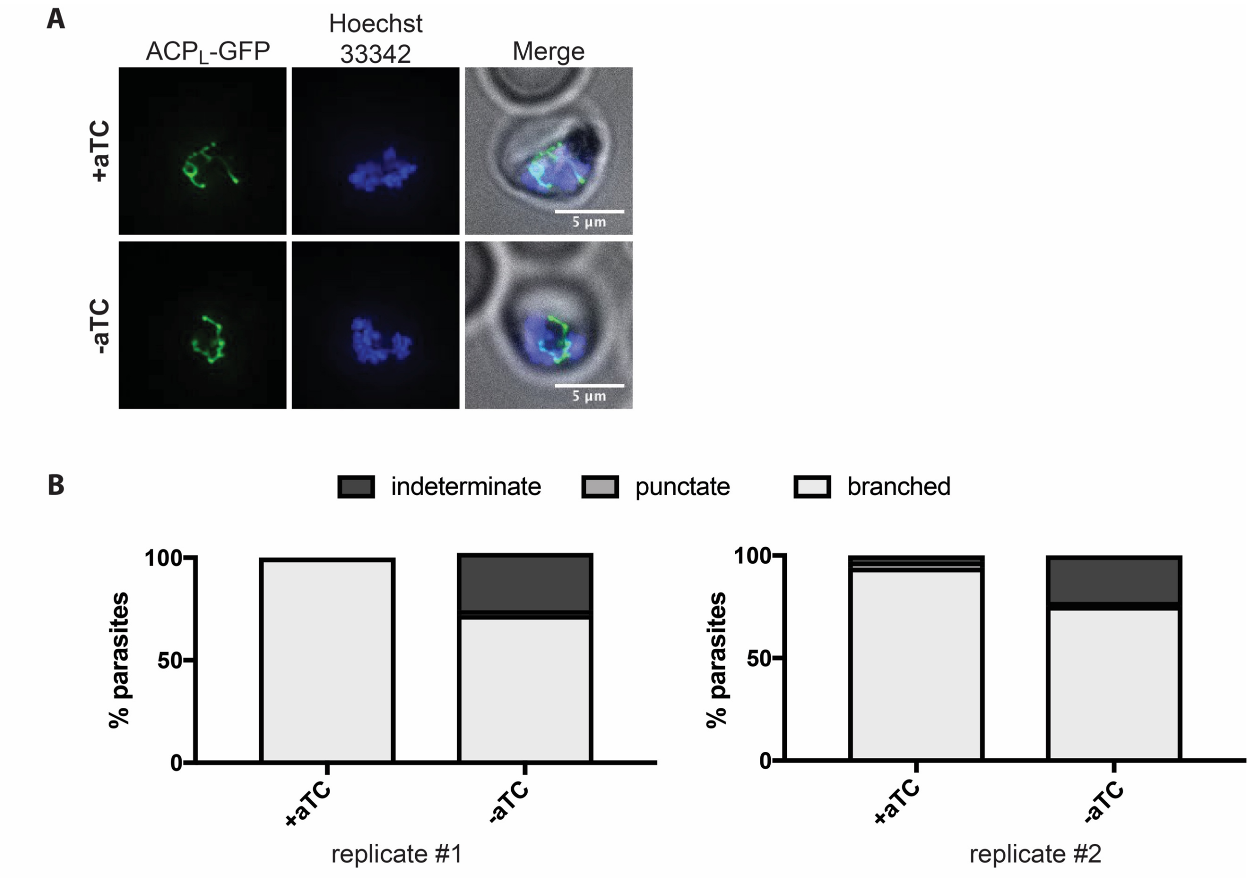

Supplementary Figure S3

(A) Representative live images of ACP_L-GFP expressing schizont stage parasites in the first cycle of ATG8 knockdown, i.e. approx. 32 hrs after aTC removal. Scale bar 5 µm. (B) Separate biological replicates of the experiment shown in Figure 3B. Thirteen and 43 parasites for replicate #1, and 34 and 55 parasites for replicate 2 were counted for +aTC and –aTC condition, respectively.
