# Supplementary material for: ATG8 Is Essential Specifically for an Autophagy-Independent Function in Apicoplast Biogenesis in Blood-Stage Malaria Parasites
Source: mBio. 2018 Jan 2;9(1):e02021-17. doi: 10.1128/mBio.02021-17 (PMC5750400; doi:10.1128/mBio.02021-17)
Supplement: FIG S4 [file mbo001183655sf4.pdf]

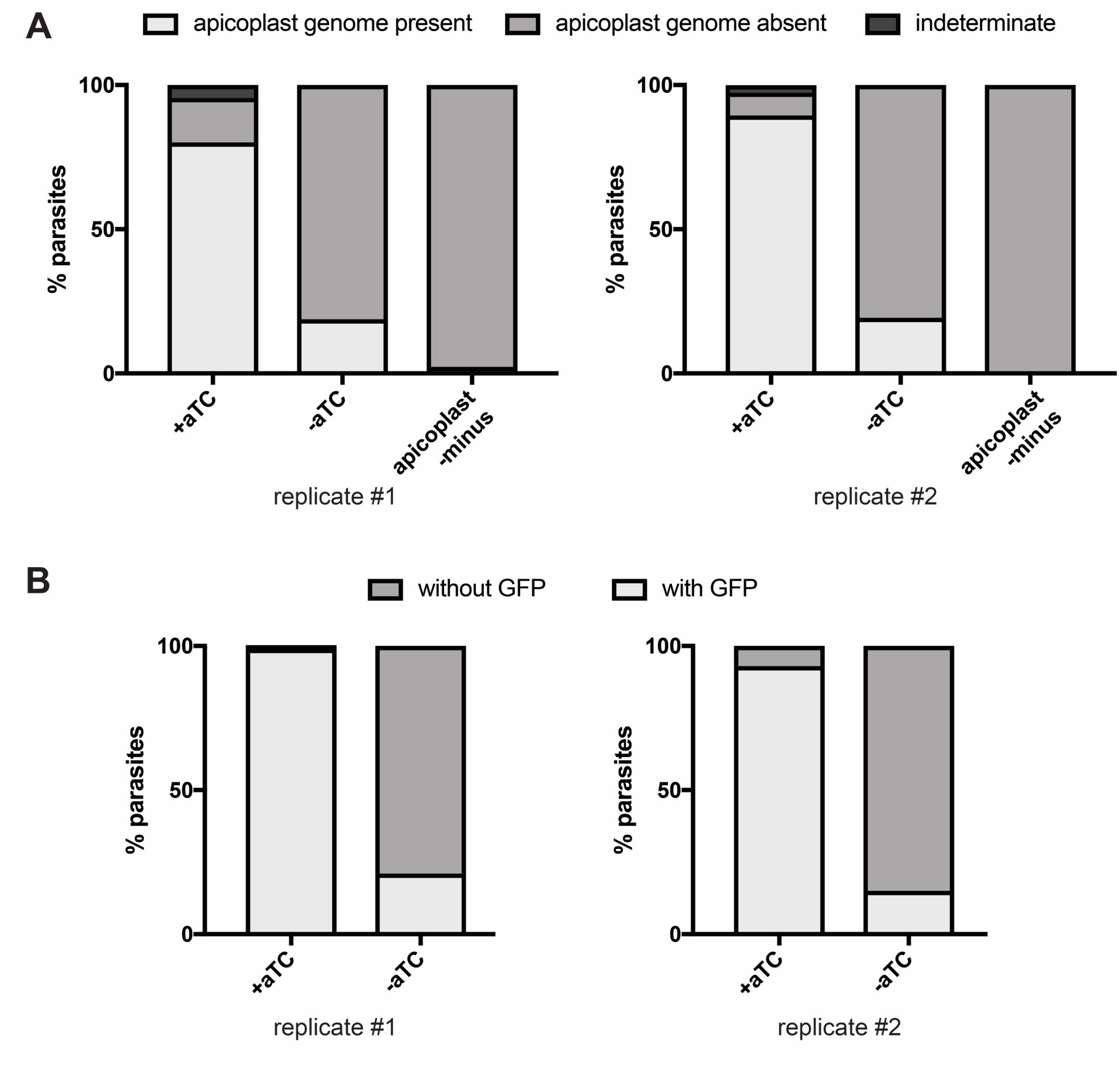

Supplementary Figure S4

(A) Separate biological replicates of the experiment shown in Figure 4A-B. Minimum 110 parasites were counted per condition per single experiment. (B) Separate biological replicates of the experiment shown in Figure 4C-D. Minimum 68 parasites were counted per condition per single experiment.
