# Supplementary material for: ATG8 Is Essential Specifically for an Autophagy-Independent Function in Apicoplast Biogenesis in Blood-Stage Malaria Parasites
Source: mBio. 2018 Jan 2;9(1):e02021-17. doi: 10.1128/mBio.02021-17 (PMC5750400; doi:10.1128/mBio.02021-17)
Supplement: TABLE S1 [file mbo001183655st1.pdf]

Supplementary Table 1.

List of primers used in this study.

**SMG413**	TTATTGGTTTTCAAACTTCATTGACTGTGCCCATTT AGGTTATTTTATGACTCTTTGTC	ATG8 Left homology region, forward
**SMG425**	TTCCGTACGGCCCAAACAAGATTCACAGCTATATT CCATATATAAATATCCATCTTCATC	ATG8 Left homology region, reverse
**SMG411**	GCATACATTATACGAAGTTATCGTAACTATAACAC GCGCACACACACACACACAC	ATG8 Right homology region forward
**SMG412**	ATAGTGAGTCGTATTATTCGCTACCTTAGGACCGT TGTGTATAAACACAAAATGAAGTATTATATATG	ATG8 Right homology region, reverse
**SMG454**	TACACATGAATATATATTTTTTGTGAGAG	ATG8 Forward for checking 5' integration
**SMG419**	CTAAGGTAGCGAATAATACGACTCACTATAGGTAT AGTTGTGAGAGTTGTCTGTTTTAGA	ATG8 gRNA primer 1
**SMG420**	CTAGCCTTATTTTAACTTGCTATTTCTAGCTCTAAA ACAGACAACTCTCACAACTATACC	ATG8 gRNA primer 2
**SMG530**	TAA TAT GAA TGA CAA AAA AGG AGA AAA TGA AG	ATG8 For checking 3' integration (P4)
**MAWA059**	TTACAAAGTATCCTAGGATGAAGATCTTATTACTT TGTATAATTTTTC	ACPL-GFP forward
**MAWA060**	TTTAATGGGGTACCCGCGGTTATTTGTATAGTTCAT CCATGCCATGTG	ACPL-GFP reverse
